# Supplementary material for: Social fluidity mobilizes contagion in human and animal populations
Source: eLife. 2021 Jul 30;10:e62177. doi: 10.7554/eLife.62177 (PMC8324292; doi:10.7554/eLife.62177)
Supplement: Supplementary file 1. [file elife-62177-supp1.pdf]

Social fluidity mobilizes contagion in human and animal populations: supplementary tables

Ewan Colman[1,2], Vittoria Colizza[3], Ephraim M. Hanks[4], David P. Hughes[5], and Shweta Bansal[1]

[1]Department of Biology, Georgetown University, Washington DC, United States
[2]Roslin Institute, University of Edinburgh, Easter Bush, Midlothian, United Kingdom
[3]INSERM, Sorbonne Université, Institut Pierre Louis d'Épidémiologie et de Santé Publique (IPLESP UMRS 1136), F75012, Paris, France.
[4]Department of Statistics, Eberly College of Science, Penn State University, State College, United States
[5]Department of Entomology, College of Agricultural Sciences, Penn State University, State College, United States

S1 Correlations between social variables

The Spearman correlation coefficient calculated across all networks, and for subsets of networks grouped by their source. Only correlations with $p < 0.05$ are shown.

All networks (57 networks)

	N	$\bar{k}$	$\bar{s}$	$\bar{w}$	$\sigma_w^2/\bar{w}$	$\bar{k}+\sigma_k^2/\bar{k}$	Q	$\bar{C}$	ϕ
Population size, N	1.00		−0.50	−0.39	−0.27		0.64	−0.74	
Mean degree, $\bar{k}$		1.00	0.69			0.93	−0.43	0.57	0.46
Mean strength, $\bar{s}$	−0.50	0.69	1.00	0.75	0.54	0.53		0.82	
Mean edge weight, $\bar{w}$	−0.39		0.75	1.00	0.88			0.59	−0.67
Edge weight heterogeneity, $\sigma_w^2/\bar{w}$	−0.27		0.54	0.88	1.00		0.30	0.33	−0.68
Excess degree, $\bar{k}+\sigma_k^2/\bar{k}$		0.93	0.53			1.00	−0.31	0.34	0.46
Modularity, Q	0.64	−0.43			0.30	−0.31	1.00	−0.39	−0.72
Mean clustering, $\bar{C}$	−0.74	0.57	0.82	0.59	0.33	0.34	−0.39	1.00	
Social fluidity, ϕ		0.46		−0.67	−0.68	0.46	−0.72		1.00

Conference Face-to-face (3 networks)

	N	$\bar{k}$	$\bar{s}$	$\bar{w}$	$\sigma_w^2/\bar{w}$	$\bar{k}+\sigma_k^2/\bar{k}$	Q	$\bar{C}$	ϕ
Population size, N	1.00	1.00	1.00	−1.00	−1.00	1.00	−1.00	1.00	1.00
Mean degree, $\bar{k}$	1.00	1.00	1.00	−1.00	−1.00	1.00	−1.00	1.00	1.00
Mean strength, $\bar{s}$	1.00	1.00	1.00	−1.00	−1.00	1.00	−1.00	1.00	1.00
Mean edge weight, $\bar{w}$	−1.00	−1.00	−1.00	1.00	1.00	−1.00	1.00	−1.00	−1.00
Edge weight heterogeneity, $\sigma_w^2/\bar{w}$	−1.00	−1.00	−1.00	1.00	1.00	−1.00	1.00	−1.00	−1.00
Excess degree, $\bar{k}+\sigma_k^2/\bar{k}$	1.00	1.00	1.00	−1.00	−1.00	1.00	−1.00	1.00	1.00
Modularity, Q	−1.00	−1.00	−1.00	1.00	1.00	−1.00	1.00	−1.00	−1.00
Mean clustering, $\bar{C}$	1.00	1.00	1.00	−1.00	−1.00	1.00	−1.00	1.00	1.00
Social fluidity, ϕ	1.00	1.00	1.00	−1.00	−1.00	1.00	−1.00	1.00	1.00

Hospital Face-to-face (4 networks)

	N	$\bar{k}$	$\bar{s}$	$\bar{w}$	$\sigma_w^2/\bar{w}$	$\bar{k}+\sigma_k^2/\bar{k}$	Q	$\bar{C}$	ϕ
Population size, N	1.00								
Mean degree, $\bar{k}$		1.00	1.00						
Mean strength, $\bar{s}$		1.00	1.00						
Mean edge weight, $\bar{w}$				1.00					−1.00
Edge weight heterogeneity, $\sigma_w^2/\bar{w}$					1.00	−1.00	1.00		
Excess degree, $\bar{k}+\sigma_k^2/\bar{k}$					−1.00	1.00	−1.00		
Modularity, Q					1.00	−1.00	1.00		
Mean clustering, $\bar{C}$								1.00	
Social fluidity, ϕ				−1.00					1.00

High school Face-to-face (5 networks)

	N	$\bar{k}$	$\bar{s}$	$\bar{w}$	$\sigma_w^2/\bar{w}$	$\bar{k}+\sigma_k^2/\bar{k}$	Q	$\bar{C}$	ϕ
Population size, N	1.00	1.00	1.00		0.90	0.90	−0.90	0.90	1.00
Mean degree, $\bar{k}$	1.00	1.00	1.00		0.90	0.90	−0.90	0.90	1.00
Mean strength, $\bar{s}$	1.00	1.00	1.00		0.90	0.90	−0.90	0.90	1.00
Mean edge weight, $\bar{w}$				1.00					
Edge weight heterogeneity, $\sigma_w^2/\bar{w}$	0.90	0.90	0.90		1.00	1.00		1.00	0.90
Excess degree, $\bar{k}+\sigma_k^2/\bar{k}$	0.90	0.90	0.90		1.00	1.00		1.00	0.90
Modularity, Q	−0.90	−0.90	−0.90				1.00		−0.90
Mean clustering, $\bar{C}$	0.90	0.90	0.90		1.00	1.00		1.00	0.90
Social fluidity, ϕ	1.00	1.00	1.00		0.90	0.90	−0.90	0.90	1.00

Ant Food sharing (6 networks)

	N	$\bar{k}$	$\bar{s}$	$\bar{w}$	$\sigma_w^2/\bar{w}$	$\bar{k}+\sigma_k^2/\bar{k}$	Q	$\bar{C}$	ϕ
Population size, N	1.00				0.94				
Mean degree, $\bar{k}$		1.00	1.00			0.94	−0.94		
Mean strength, $\bar{s}$		1.00	1.00			0.94	−0.94		
Mean edge weight, $\bar{w}$				1.00				0.89	
Edge weight heterogeneity, $\sigma_w^2/\bar{w}$	0.94				1.00				
Excess degree, $\bar{k}+\sigma_k^2/\bar{k}$		0.94	0.94			1.00	−0.89	0.83	0.83
Modularity, Q		−0.94	−0.94			−0.89	1.00	−0.89	
Mean clustering, $\bar{C}$				0.89		0.83	−0.89	1.00	
Social fluidity, ϕ						0.83			1.00

Ant Antennal contact (6 networks)

	N	$\bar{k}$	$\bar{s}$	$\bar{w}$	$\sigma_w^2/\bar{w}$	$\bar{k}+\sigma_k^2/\bar{k}$	Q	$\bar{C}$	ϕ
Population size, N	1.00								
Mean degree, $\bar{k}$		1.00	0.94						
Mean strength, $\bar{s}$		0.94	1.00	0.89	0.89				
Mean edge weight, $\bar{w}$			0.89	1.00	1.00				−0.83
Edge weight heterogeneity, $\sigma_w^2/\bar{w}$			0.89	1.00	1.00				−0.83
Excess degree, $\bar{k}+\sigma_k^2/\bar{k}$						1.00			
Modularity, Q							1.00		
Mean clustering, $\bar{C}$								1.00	
Social fluidity, ϕ				−0.83	−0.83				1.00

Bee Food sharing (5 networks)

	N	$\bar{k}$	$\bar{s}$	$\bar{w}$	$\sigma_w^2/\bar{w}$	$\bar{k}+\sigma_k^2/\bar{k}$	Q	$\bar{C}$	ϕ
Population size, N	1.00	0.90	0.90			0.90	−1.00	0.90	0.90
Mean degree, $\bar{k}$	0.90	1.00	1.00			1.00	−0.90	1.00	
Mean strength, $\bar{s}$	0.90	1.00	1.00			1.00	−0.90	1.00	
Mean edge weight, $\bar{w}$				1.00	0.90				
Edge weight heterogeneity, $\sigma_w^2/\bar{w}$				0.90	1.00				−0.90
Excess degree, $\bar{k}+\sigma_k^2/\bar{k}$	0.90	1.00	1.00			1.00	−0.90	1.00	
Modularity, Q	−1.00	−0.90	−0.90			−0.90	1.00	−0.90	−0.90
Mean clustering, $\bar{C}$	0.90	1.00	1.00			1.00	−0.90	1.00	
Social fluidity, ϕ	0.90				−0.90		−0.90		1.00

Parakeet Aggression (8 networks)

	N	$\bar{k}$	$\bar{s}$	$\bar{w}$	$\sigma_w^2/\bar{w}$	$\bar{k}+\sigma_k^2/\bar{k}$	Q	$\bar{C}$	ϕ
Population size, N	1.00						0.87	−0.76	
Mean degree, $\bar{k}$		1.00				1.00		0.81	
Mean strength, $\bar{s}$			1.00	0.95				0.83	−0.74
Mean edge weight, $\bar{w}$			0.95	1.00				0.83	−0.81
Edge weight heterogeneity, $\sigma_w^2/\bar{w}$					1.00				
Excess degree, $\bar{k}+\sigma_k^2/\bar{k}$		1.00				1.00		0.81	
Modularity, Q	0.87						1.00		
Mean clustering, $\bar{C}$	−0.76	0.81	0.83	0.83		0.81		1.00	
Social fluidity, ϕ			−0.74	−0.81					1.00

Shark Association (6 networks)

	N	$\bar{k}$	$\bar{s}$	$\bar{w}$	$\sigma_w^2/\bar{w}$	$\bar{k}+\sigma_k^2/\bar{k}$	Q	$\bar{C}$	ϕ
Population size, N	1.00	1.00	0.94			0.94			
Mean degree, $\bar{k}$	1.00	1.00	0.94			0.94			
Mean strength, $\bar{s}$	0.94	0.94	1.00	0.83	0.83	0.83			
Mean edge weight, $\bar{w}$			0.83	1.00	1.00				
Edge weight heterogeneity, $\sigma_w^2/\bar{w}$			0.83	1.00	1.00				
Excess degree, $\bar{k}+\sigma_k^2/\bar{k}$	0.94	0.94	0.83			1.00			
Modularity, Q							1.00	0.94	−0.89
Mean clustering, $\bar{C}$							0.94	1.00	
Social fluidity, ϕ							−0.89		1.00

S2 Correlations between social variables and simulated basic reproduction number

The Pearson correlation coefficient between quantities calculated on the network and the simulated disease outcomes for $R_0^\infty = 2$, 3, and 4, and for synthetic interaction time (for networks for which real interacting times are not available) generated with Poisson, bursty, and circadian interaction time series. Correlation between the error, the predicted and mean simulated basic reproduction number, $\|R_0^\phi - R_0^{sim}\|$ of are also presented. Only correlations with $p < 0.05$ are shown.

$R^* = 2$, time series: Poisson

	Correlation with R_0^{sim}			Corr. with prediction error		
	$g = 0$	$g = 1$	$g = 2$	$g = 0$	$g = 1$	$g = 2$
Estimated rep. number, R_0^{Est}	0.72	0.87	0.87			
Mean individual rep. number, $\bar{r}(s_i)$	0.96	0.67	0.61	N/A	N/A	N/A
Social fluidity, ϕ	0.72	0.72	0.63	−0.69	−0.44	
Network size, N		0.37	0.50			−0.45
Excess degree, $\bar{k} + \sigma_k^2/\bar{k}$	0.58	0.67	0.64			−0.29
Mean degree, $\bar{k}$	0.68	0.57	0.51			
Mean strength, $\bar{s}$				0.44		
Mean edge weight, $\bar{w}$		−0.38	−0.50	0.68	0.39	
Edge weight heterogeneity, $\sigma_w^2/\bar{w}$	−0.30	−0.45	−0.53	0.58	0.37	
Modularity, Q	−0.64	−0.63	−0.44	0.47	0.36	
Mean clustering, $\bar{C}$	0.28			0.29		0.33
Mean prediction error (%)	11.2	9.0	11.4			

$R^* = 2$, time series: Circadian

	Correlation with R_0^{sim}			Corr. with prediction error		
	$g = 0$	$g = 1$	$g = 2$	$g = 0$	$g = 1$	$g = 2$
Estimated rep. number, R_0^{Est}	0.73	0.86	0.87			
Mean individual rep. number, $\bar{r}(s_i)$	0.96	0.71	0.59	N/A	N/A	N/A
Social fluidity, ϕ	0.72	0.71	0.61	−0.70	−0.50	
Network size, N		0.36	0.53			−0.48
Excess degree, $\bar{k} + \sigma_k^2/\bar{k}$	0.56	0.69	0.62			−0.30
Mean degree, $\bar{k}$	0.65	0.61	0.49			
Mean strength, $\bar{s}$				0.42	0.30	
Mean edge weight, $\bar{w}$		−0.34	−0.48	0.71	0.51	
Edge weight heterogeneity, $\sigma_w^2/\bar{w}$	−0.30	−0.45	−0.51	0.58	0.45	
Modularity, Q	−0.65	−0.65	−0.44	0.47	0.29	
Mean clustering, $\bar{C}$	0.29		−0.26	0.34	0.29	0.33
Mean prediction error (%)	12.5	9.4	11.5			

$R^* = 2$, time series: Bursty

	Correlation with R_0^{sim}			Corr. with prediction error		
	$g = 0$	$g = 1$	$g = 2$	$g = 0$	$g = 1$	$g = 2$
Estimated rep. number, R_0^{Est}	0.73	0.88	0.87			
Mean individual rep. number, $\bar{r}(s_i)$	0.96	0.72	0.59	N/A	N/A	N/A
Social fluidity, ϕ	0.71	0.76	0.61	−0.73	−0.47	
Network size, N		0.34	0.53			−0.41
Excess degree, $\bar{k} + \sigma_k^2/\bar{k}$	0.58	0.66	0.62			
Mean degree, $\bar{k}$	0.67	0.58	0.48			
Mean strength, $\bar{s}$				0.38	0.28	
Mean edge weight, $\bar{w}$		−0.38	−0.47	0.67	0.47	
Edge weight heterogeneity, $\sigma_w^2/\bar{w}$	−0.30	−0.47	−0.50	0.57	0.40	
Modularity, Q	−0.62	−0.68	−0.45	0.51	0.29	
Mean clustering, $\bar{C}$	0.27		−0.28		0.29	0.36
Mean prediction error (%)	11.9	9.0	11.4			

$R^* = 3$, time series: Poisson

	Correlation with R_0^{sim}			Corr. with prediction error		
	$g = 0$	$g = 1$	$g = 2$	$g = 0$	$g = 1$	$g = 2$
Estimated rep. number, R_0^{Est}	0.77	0.91	0.87			
Mean individual rep. number, $\bar{r}(s_i)$	0.96	0.73	0.59	N/A	N/A	N/A
Social fluidity, ϕ	0.74	0.73	0.55	−0.73	−0.28	
Network size, N		0.47	0.67			−0.60
Excess degree, $\bar{k} + \sigma_k^2/\bar{k}$	0.60	0.64	0.53			−0.31
Mean degree, $\bar{k}$	0.67	0.53	0.37			
Mean strength, $\bar{s}$				0.42		
Mean edge weight, $\bar{w}$		−0.45	−0.52	0.64	0.30	
Edge weight heterogeneity, $\sigma_w^2/\bar{w}$	−0.34	−0.48	−0.49	0.48	0.28	
Modularity, Q	−0.63	−0.59	−0.33	0.46	0.28	−0.46
Mean clustering, $\bar{C}$			−0.43			0.28
Mean prediction error (%)	11.9	8.8	18.0			

$R^* = 3$, time series: Circadian

	Correlation with R_0^{sim}			Corr. with prediction error		
	$g = 0$	$g = 1$	$g = 2$	$g = 0$	$g = 1$	$g = 2$
Estimated rep. number, R_0^{Est}	0.79	0.92	0.87			
Mean individual rep. number, $\bar{r}(s_i)$	0.96	0.73	0.60	N/A	N/A	N/A
Social fluidity, ϕ	0.76	0.72	0.55	−0.69		
Network size, N		0.48	0.66			−0.63
Excess degree, $\bar{k} + \sigma_k^2/\bar{k}$	0.61	0.62	0.53			−0.35
Mean degree, $\bar{k}$	0.68	0.51	0.37			
Mean strength, $\bar{s}$				0.42		
Mean edge weight, $\bar{w}$		−0.46	−0.53	0.61	0.29	
Edge weight heterogeneity, $\sigma_w^2/\bar{w}$	−0.38	−0.48	−0.48	0.45	0.27	
Modularity, Q	−0.65	−0.59	−0.34	0.47	0.28	−0.45
Mean clustering, $\bar{C}$			−0.45	0.29		0.32
Mean prediction error (%)	11.7	8.6	17.6			

$R^* = 3$, time series: Bursty

	Correlation with R_0^{sim}			Corr. with prediction error		
	$g=0$	$g=1$	$g=2$	$g=0$	$g=1$	$g=2$
Estimated rep. number, R_0^{Est}	0.78	0.90	0.86			
Mean individual rep. number, $\bar{r}(s_i)$	0.96	0.71	0.59	N/A	N/A	N/A
Social fluidity, ϕ	0.75	0.71	0.54	−0.69		0.27
Network size, N		0.47	0.69			−0.64
Excess degree, $\bar{k} + \sigma_k^2/\bar{k}$	0.62	0.63	0.52			−0.32
Mean degree, $\bar{k}$	0.69	0.51	0.37			
Mean strength, $\bar{s}$				0.49		
Mean edge weight, $\bar{w}$		−0.45	−0.50	0.68		
Edge weight heterogeneity, $\sigma_w^2/\bar{w}$	−0.36	−0.48	−0.45	0.52		
Modularity, Q	−0.64	−0.57	−0.32	0.48		−0.48
Mean clustering, $\bar{C}$			−0.45	0.34		0.27
Mean prediction error (%)	11.6	9.3	17.8			

$R^* = 4$, time series: Poisson

	Correlation with R_0^{sim}			Corr. with prediction error		
	$g=0$	$g=1$	$g=2$	$g=0$	$g=1$	$g=2$
Estimated rep. number, R_0^{Est}	0.84	0.92	0.84			
Mean individual rep. number, $\bar{r}(s_i)$	0.96	0.71	0.56	N/A	N/A	N/A
Social fluidity, ϕ	0.76	0.66	0.45	−0.80		0.33
Network size, N	0.30	0.60	0.77		−0.40	−0.76
Excess degree, $\bar{k} + \sigma_k^2/\bar{k}$	0.64	0.56	0.42			
Mean degree, $\bar{k}$	0.68	0.43				
Mean strength, $\bar{s}$			−0.29	0.39		
Mean edge weight, $\bar{w}$	−0.28	−0.49	−0.53	0.68		
Edge weight heterogeneity, $\sigma_w^2/\bar{w}$	−0.41	−0.46	−0.45	0.54		
Modularity, Q	−0.63	−0.49		0.60		−0.62
Mean clustering, $\bar{C}$		−0.32	−0.57		0.27	0.49
Mean prediction error (%)	11.3	10.6	28.9			

$R^* = 4$, time series: Circadian

	Correlation with R_0^{sim}			Corr. with prediction error		
	$g=0$	$g=1$	$g=2$	$g=0$	$g=1$	$g=2$
Estimated rep. number, R_0^{Est}	0.83	0.91	0.83			
Mean individual rep. number, $\bar{r}(s_i)$	0.97	0.71	0.55	N/A	N/A	N/A
Social fluidity, ϕ	0.77	0.65	0.45	−0.81		0.32
Network size, N		0.59	0.77		−0.42	−0.75
Excess degree, $\bar{k} + \sigma_k^2/\bar{k}$	0.64	0.57	0.42			
Mean degree, $\bar{k}$	0.68	0.43				
Mean strength, $\bar{s}$			−0.28	0.35		
Mean edge weight, $\bar{w}$	−0.29	−0.49	−0.53	0.65		
Edge weight heterogeneity, $\sigma_w^2/\bar{w}$	−0.41	−0.46	−0.44	0.52		
Modularity, Q	−0.63	−0.48		0.62		−0.61
Mean clustering, $\bar{C}$		−0.32	−0.56		0.33	0.45
Mean prediction error (%)	10.3	10.6	29.0			

$R^* = 4$, time series: Bursty

	Correlation with R_0^{sim}			Corr. with prediction error		
	$g=0$	$g=1$	$g=2$	$g=0$	$g=1$	$g=2$
Estimated rep. number, R_0^{Est}	0.83	0.93	0.83			
Mean individual rep. number, $\bar{r}(s_i)$	0.96	0.73	0.55	N/A	N/A	N/A
Social fluidity, ϕ	0.76	0.68	0.45	-0.81		0.32
Network size, N	0.28	0.59	0.77		-0.45	-0.72
Excess degree, $\bar{k} + \sigma_k^2/\bar{k}$	0.63	0.57	0.42			
Mean degree, $\bar{k}$	0.68	0.44				
Mean strength, $\bar{s}$			-0.27	0.31		
Mean edge weight, $\bar{w}$	-0.29	-0.49	-0.52	0.62		
Edge weight heterogeneity, $\sigma_w^2/\bar{w}$	-0.42	-0.46	-0.42	0.48		
Modularity, Q	-0.61	-0.50		0.70		-0.57
Mean clustering, $\bar{C}$		-0.31	-0.56		0.30	0.44
Mean prediction error (%)	11.1	10.2	28.4			
