# Supplementary material for: Social fluidity mobilizes contagion in human and animal populations
Source: eLife. 2021 Jul 30;10:e62177. doi: 10.7554/eLife.62177 (PMC8324292; doi:10.7554/eLife.62177)
Supplement: Supplementary file 2. [file elife-62177-supp2.zip › Results_for_all_networks.csv]

Network	Species	Interaction	phi	epsilon	population	degree	excess_deg	mean_stre
ants_1_hig	Ant	Food sharir	0.97	2.06E-03	74	7.92	10.68	13.41
ants_2_hig	Ant	Food sharir	0.94	2.11E-03	68	5.76	8.53	9.35
ants_3_hig	Ant	Food sharir	1.12	2.80E-03	76	10.5	15.03	17.74
ants_1_low	Ant	Food sharir	1.12	3.10E-03	70	10.17	14.53	17.31
ants_2_low	Ant	Food sharir	0.94	1.74E-03	79	8.2	10.6	13.85
ants_3_low	Ant	Food sharir	0.95	1.76E-03	82	8.34	12.57	14.88
conference	Conference	Face-to-fac	0.66	4.23E-04	93	5.63	10.43	14.26
conference	Conference	Face-to-fac	0.51	1.37E-04	92	5.2	8.38	14.13
conference	Conference	Face-to-fac	0.24	1.18E-06	84	3.19	4.72	11.36
hospital_0	Hospital	Face-to-fac	0.61	8.42E-04	51	8.35	12.2	30.51
hospital_1	Hospital	Face-to-fac	0.58	7.52E-04	49	10.2	13.69	43.88
hospital_2	Hospital	Face-to-fac	0.68	1.24E-03	51	9.49	14.67	33.53
hospital_3	Hospital	Face-to-fac	0.58	7.42E-04	50	7.92	11.6	29.72
school_0	Primary sch	Face-to-fac	0.61	6.64E-05	237	14.72	17.37	54.18
school_1	Primary sch	Face-to-fac	0.62	6.97E-05	238	14.87	18.19	54.74
highschool_	High schoo	Face-to-fac	0.4	1.66E-06	312	6.78	9.14	31.53
highschool_	High schoo	Face-to-fac	0.39	1.30E-06	311	6.41	8.59	28.42
highschool_	High schoo	Face-to-fac	0.32	2.15E-07	299	4.99	6.77	22.84
highschool_	High schoo	Face-to-fac	0.31	1.33E-07	294	4.98	6.81	22.66
highschool_	High schoo	Face-to-fac	0.17	1.30E-10	233	2.71	3.91	9.3
office_0	Office	Face-to-fac	0.08	1.52E-09	46	2.13	2.86	5.3
antennatio	Ant	Antennal c	0.81	9.60E-04	89	14.58	18.56	38.81
antennatio	Ant	Antennal c	0.8	1.24E-03	72	15.44	19.16	44.5
antennatio	Ant	Antennal c	1.17	3.33E-03	71	14.23	18.92	25.92
antennatio	Ant	Antennal c	0.78	1.21E-03	69	15.91	21.19	52.93
antennatio	Ant	Antennal c	1.2	8.45E-03	33	15.64	17.62	38.97
antennatio	Ant	Antennal c	1.08	7.35E-03	32	10.12	12.57	21.81
bees_1	Bee	Food sharir	1.19	1.73E-04	1148	11.38	15.79	13.5
bees_2	Bee	Food sharir	1.03	1.08E-04	1112	8.12	11.06	10.37
bees_3	Bee	Food sharir	1.03	1.13E-04	1072	5.13	7.65	6.09
bees_4	Bee	Food sharir	1.33	2.29E-04	1160	10.86	15.55	12.09
bees_5	Bee	Food sharir	1.36	2.38E-04	1161	13.52	18.31	15.1
bats_0	Bat	Food sharir	0.39	2.50E-03	20	5.6	7.98	33.7
parakeet_1	Parakeet	Aggression	1.72	2.24E-02	21	9.33	10.47	16.67
parakeet_1	Parakeet	Aggression	1.42	1.81E-02	21	10.48	11.51	21.14
parakeet_1	Parakeet	Aggression	0.88	8.73E-03	21	10.19	11.4	30.95
parakeet_1	Parakeet	Aggression	1.77	2.30E-02	21	12.48	13.25	27.71
parakeet_2	Parakeet	Aggression	1.24	1.72E-02	19	11.89	12.74	42
parakeet_2	Parakeet	Aggression	1.35	1.91E-02	19	11.05	11.85	28.74
parakeet_2	Parakeet	Aggression	1.3	1.83E-02	19	10.42	11.4	26
parakeet_2	Parakeet	Aggression	1.18	1.60E-02	19	13.26	13.56	46.42
Bison_dom	Bison	Aggression	1.08	9.43E-03	26	17.08	18.44	69
Sheep_dor	Sheep	Aggression	1.35	1.22E-02	28	16.79	18.24	47
macaque_	Monkey	Aggression	1.87	7.73E-03	62	37.65	39.1	78.55

Howler_mo	Monkey	Associatior	0.54	4.65E-03	17	10	10.16	59.06
Massen_M	Monkey	Associatior	0.52	1.52E-03	28	16.29	17.18	144.64
cattle_dom	Cattle	Aggression	1.35	1.22E-02	28	14.64	16.3	35.57
Macaques_	Monkey	Grooming	0.37	2.26E-03	16	8.62	9.49	80.88
stumptaile	Monkey	Grooming	0.6	4.73E-03	19	11.89	12.5	78.11
Kangaroo_	Kangaroo	Associatior	0.54	4.66E-03	17	11.53	12.79	142.59
Swallow_p	Swallow	Associatior	1.08	1.62E-02	17	14.35	14.61	104.24
shark_0	Shark	Associatior	0.61	2.85E-03	26	6.08	7.35	16.54
shark_1	Shark	Associatior	0.52	4.26E-04	54	12.96	14.2	74.07
shark_2	Shark	Associatior	0.54	3.33E-04	65	13.14	15.64	69.17
shark_3	Shark	Associatior	0.55	3.53E-04	67	14.27	17.11	84.39
shark_4	Shark	Associatior	0.55	5.55E-04	53	12.45	15.4	65.43
shark_5	Shark	Associatior	0.52	5.04E-04	50	11.88	14.02	63.24

mean_wei	weight_he	modularity	within_bet	clustering	r_mean_R	R0^Est_R*	R0^Sim_R*	R0^Sim_R*
1.69	0.89	0.29	1.54	0.3	1.1	1.47	1.24	1.51
1.62	0.8	0.29	1.13	0.22	0.92	1.37	1	1.54
1.69	0.9	0.19	1.29	0.31	1.03	1.51	1.1	1.64
1.7	0.83	0.18	1.11	0.35	0.97	1.45	1.07	1.54
1.69	1.22	0.27	1.16	0.29	1.03	1.37	1.14	1.37
1.78	1.1	0.23	1.21	0.34	0.89	1.36	0.99	1.49
2.53	3.57	0.56	2.29	0.26	0.67	1.17	0.75	1.27
2.72	3.74	0.67	2.66	0.25	0.81	1.09	0.97	1.1
3.56	6.37	0.79	2.97	0.22	0.64	0.87	0.69	0.74
3.65	9.18	0.46	2.84	0.4	0.65	1.03	0.79	1.3
4.3	8.82	0.43	2.59	0.45	0.73	1.04	0.86	1.29
3.53	5.99	0.42	2.72	0.44	0.74	1.2	0.92	1.55
3.75	9.44	0.46	3.01	0.4	0.67	1.04	0.76	1.18
3.68	5.82	0.71	1.73	0.42	1.1	1.29	1.35	1.42
3.68	5.92	0.7	1.7	0.44	1.03	1.25	1.29	1.37
4.65	11.07	0.84	3.05	0.36	0.82	1.05	0.91	0.99
4.43	10.55	0.83	3.19	0.35	0.79	1	0.96	1.01
4.58	8.51	0.85	3.21	0.31	0.74	0.96	0.84	0.96
4.55	9.01	0.85	3.76	0.32	0.76	0.96	0.82	1.03
3.43	3.76	0.89	2.76	0.24	0.69	0.91	0.82	0.73
2.49	2.46	0.75	2	0.11	0.71	0.88	0.83	0.66
2.66	2.25	0.44	1.93	0.43	1.16	1.42	1.32	1.56
2.88	2.76	0.41	1.95	0.52	1.14	1.37	1.27	1.43
1.82	0.86	0.25	1.55	0.34	1.23	1.63	1.3	1.7
3.33	2.85	0.34	1.63	0.5	1.02	1.35	1.2	1.55
2.49	1.19	0.22	1.68	0.64	1.34	1.53	1.45	1.6
2.15	0.89	0.31	1.8	0.61	1.2	1.47	1.28	1.52
1.19	0.32	0.32	1.49	0.02	1.31	1.81	1.38	1.8
1.28	0.45	0.41	1.66	0.02	1.27	1.7	1.29	1.7
1.19	0.45	0.51	1.36	0.01	1.13	1.65	1.19	1.56
1.11	0.18	0.31	1.3	0.02	1.31	1.88	1.21	1.85
1.12	0.2	0.27	1.33	0.02	1.4	1.89	1.42	1.88
6.02	6.34	-	-	-	-	-	-	-
1.79	0.79	0.16	1.51	0.56	1.33	1.51	1.35	1.42
2.02	2.88	0.24	1.8	0.59	1.39	1.45	1.46	1.27
3.04	5.45	0.23	1.55	0.59	1.18	1.27	1.25	1.24
2.22	1.71	0.15	1.82	0.68	1.4	1.52	1.52	1.42
3.53	9.04	0.13	1.62	0.76	1.02	1.16	1.09	1.24
2.6	1.95	0.13	1.7	0.69	1.23	1.36	1.32	1.4
2.49	1.5	0.11	1.69	0.64	1.28	1.44	1.35	1.45
3.5	2.18	0.13	1.79	0.74	1.4	1.45	1.54	1.45
4.04	2.78	0.14	1.86	0.81	1.26	1.38	1.36	1.53
2.8	1.8	0.15	1.72	0.72	1.34	1.5	1.44	1.54
2.09	0.92	0.18	1.55	0.67	1.7	1.79	1.73	1.75

5.91	4.27	0.2	1.56	0.6	1.21	1.23	1.57	1.27
8.88	22.28	0.4	4.25	0.66	1.16	1.22	1.4	1.38
2.43	1.65	0.18	1.85	0.65	1.33	1.51	1.49	1.56
9.38	13.69	0.24	3.02	0.71	0.92	1.03	1.11	1.09
6.57	11.13	0.28	2.56	0.69	1.13	1.19	1.32	1.26
12.51	23.86	0.17	2.04	0.82	0.83	0.97	0.93	1.24
7.26	6.81	0.13	1.84	0.93	1.32	1.36	1.52	1.38
2.72	1.26	0.46	0.91	0.86	0.89	1.12	1.02	1.2
5.71	4.16	0.59	1.23	0.89	0.91	1.04	1.15	1.29
5.26	2.97	0.54	1.26	0.89	0.85	1.06	1.05	1.37
5.91	4.73	0.47	1.46	0.89	0.77	0.98	0.97	1.35
5.25	2.72	0.44	1.3	0.83	0.87	1.1	1.08	1.37
5.32	3.69	0.51	1.02	0.88	0.86	1.05	1.02	1.43

R0^Sim_R*	R0^Sim_R*	R0^Sim_R*	R0^Sim_R*	R0^Sim_R*	R0^Sim_R*	R0^Sim_R*	r_mean_R*	R0^Est_R*
1.4	1.28	1.47	1.38	1.23	1.48	1.42	1.57	2.07
1.28	1.05	1.44	1.46	0.99	1.44	1.41	1.3	1.92
1.48	1.09	1.62	1.43	1.19	1.63	1.46	1.47	2.15
1.54	1.07	1.51	1.53	1.01	1.53	1.5	1.4	2.06
1.25	1.1	1.35	1.25	1.14	1.4	1.3	1.46	1.92
1.49	1.02	1.49	1.42	1.02	1.51	1.46	1.26	1.92
1.23	0.85	1.17	1.13	0.79	1.19	1.21	0.93	1.6
0.94	0.95	1.1	0.97	0.97	1.08	0.9	1.1	1.47
0.7	0.77	0.69	0.72	0.77	0.7	0.73	0.85	1.14
1.06	0.79	1.11	1.07	0.7	1.2	1.03	0.9	1.4
1.16	0.83	1.39	1.07	0.84	1.32	1.08	1.01	1.41
1.14	0.83	1.44	1.16	0.95	1.43	1.21	1.03	1.64
1.01	0.81	1.14	1.02	0.81	1.14	1.02	0.93	1.41
1.34	1.34	1.38	1.32	1.34	1.42	1.32	1.52	1.77
1.39	1.17	1.41	1.35	1.26	1.39	1.34	1.41	1.72
0.99	0.97	1.02	1.06	0.94	1.03	1	1.1	1.39
0.92	0.93	0.94	0.96	0.97	0.91	0.91	1.06	1.33
0.89	0.81	0.94	0.82	0.86	0.96	0.81	0.99	1.26
0.81	0.95	0.89	0.8	0.87	0.89	0.84	1.01	1.26
0.77	0.8	0.72	0.71	0.79	0.73	0.66	0.9	1.17
0.68	0.77	0.58	0.63	0.86	0.65	0.68	0.92	1.13
1.4	1.35	1.56	1.43	1.23	1.49	1.47	1.62	1.98
1.52	1.33	1.52	1.45	1.33	1.57	1.42	1.6	1.91
1.57	1.34	1.63	1.56	1.26	1.68	1.53	1.77	2.33
1.48	1.18	1.57	1.55	1.24	1.52	1.53	1.42	1.86
1.38	1.49	1.54	1.37	1.46	1.56	1.39	1.9	2.15
1.35	1.36	1.46	1.29	1.28	1.48	1.23	1.68	2.06
1.69	1.39	1.81	1.74	1.39	1.81	1.8	1.93	2.65
1.72	1.27	1.75	1.64	1.37	1.66	1.65	1.84	2.46
1.55	1.21	1.6	1.56	1.19	1.56	1.6	1.64	2.39
1.77	1.35	1.81	1.86	1.33	1.88	1.84	1.95	2.77
1.77	1.46	1.86	1.85	1.39	1.83	1.78	2.08	2.79
-	-	-	-	-	-	-	-	-
1.22	1.42	1.41	1.23	1.39	1.47	1.22	1.88	2.13
1.1	1.52	1.32	1.12	1.49	1.3	1.15	1.96	2.04
1.07	1.33	1.31	1.09	1.33	1.26	1.05	1.62	1.74
1.22	1.48	1.4	1.17	1.47	1.49	1.17	1.98	2.15
1.03	1.09	1.25	0.94	1.05	1.32	0.98	1.43	1.61
1.15	1.32	1.41	1.14	1.34	1.49	1.12	1.73	1.9
1.16	1.41	1.39	1.12	1.42	1.48	1.12	1.79	2
1.2	1.59	1.45	1.15	1.52	1.51	1.18	1.95	2.01
1.31	1.52	1.51	1.31	1.43	1.61	1.26	1.76	1.93
1.31	1.45	1.54	1.33	1.48	1.49	1.31	1.9	2.11
1.68	1.69	1.79	1.63	1.76	1.74	1.62	2.49	2.61

1.14	1.55	1.36	1.08	1.53	1.34	1.1	1.62	1.64
1.2	1.49	1.34	1.18	1.49	1.35	1.2	1.56	1.64
1.3	1.45	1.56	1.32	1.45	1.48	1.3	1.87	2.13
0.93	1.17	1.12	0.98	1.12	1.07	0.97	1.22	1.36
1.1	1.34	1.3	1.07	1.38	1.28	1.12	1.52	1.6
0.83	0.94	1.2	0.9	0.89	1.19	0.94	1.13	1.3
1.12	1.45	1.41	1.14	1.47	1.42	1.12	1.82	1.88
0.92	1.07	1.2	0.98	1.07	1.32	0.96	1.22	1.51
1.05	1.14	1.35	1.09	1.13	1.27	1.11	1.24	1.4
1.21	1.11	1.37	1.29	1.11	1.34	1.2	1.15	1.43
1.25	1.02	1.36	1.27	0.98	1.31	1.28	1.06	1.33
1.25	1.17	1.44	1.24	1.1	1.44	1.26	1.18	1.49
1.21	1.06	1.45	1.1	1.06	1.38	1.13	1.16	1.42

R0^Sim_R*	R0^Sim_R*	R0^Sim_R*	R0^Sim_R*	R0^Sim_R*	R0^Sim_R*	R0^Sim_R*	R0^Sim_R*	R0^Sim_R*
1.75	2.1	1.87	1.82	2.13	1.82	1.72	2.04	1.84
1.4	1.89	1.69	1.46	2.02	1.71	1.36	2.01	1.71
1.65	2.33	1.91	1.68	2.26	2.11	1.59	2.36	1.94
1.56	2.16	1.94	1.56	2.13	1.87	1.54	2.09	1.87
1.57	1.89	1.72	1.64	1.9	1.63	1.68	1.83	1.66
1.49	2.06	1.83	1.37	2.02	1.95	1.35	2.12	1.84
0.97	1.58	1.56	1	1.63	1.44	1	1.63	1.44
1.26	1.46	1.36	1.3	1.45	1.38	1.29	1.49	1.32
0.93	0.95	0.87	0.91	0.91	0.89	0.97	0.88	0.83
1	1.62	1.42	1.07	1.69	1.35	1.04	1.59	1.49
1.24	1.77	1.45	1.14	1.77	1.43	1.22	1.8	1.46
1.19	1.93	1.51	1.16	1.86	1.56	1.19	1.94	1.51
0.99	1.64	1.3	1.02	1.52	1.33	1.06	1.49	1.35
1.94	1.89	1.71	1.78	1.93	1.79	1.88	1.97	1.79
1.72	1.83	1.77	1.74	1.87	1.77	1.72	1.89	1.8
1.34	1.32	1.27	1.28	1.29	1.26	1.29	1.32	1.32
1.23	1.2	1.18	1.24	1.24	1.18	1.2	1.29	1.2
1.19	1.19	1.09	1.1	1.25	1.08	1.1	1.25	1.18
1.16	1.2	0.98	1.16	1.17	1.07	1.15	1.16	1.1
1.03	0.87	0.94	1.03	0.89	0.91	1.04	0.83	0.95
1.14	0.8	0.76	1.08	0.82	0.8	1.04	0.83	0.75
1.92	2.02	1.84	1.92	2.09	1.97	1.95	2.11	1.9
1.86	2.08	1.88	1.83	2.04	1.89	1.86	2.09	1.8
1.97	2.27	2.04	1.84	2.38	2.08	2.02	2.3	2.02
1.64	2.07	1.89	1.64	2.04	1.97	1.7	2.04	1.96
2.06	2.02	1.68	2.12	1.99	1.67	2.18	2.04	1.63
1.86	1.95	1.64	1.78	1.99	1.62	1.95	1.95	1.59
2.08	2.68	2.6	2.09	2.68	2.65	1.96	2.65	2.62
2.06	2.43	2.41	1.95	2.47	2.51	1.93	2.51	2.48
1.69	2.2	2.28	1.73	2.24	2.23	1.72	2.32	2.28
1.91	2.82	2.69	2.06	2.69	2.6	2	2.58	2.68
2.13	2.75	2.62	2.11	2.71	2.66	2.16	2.72	2.65
-	-	-	-	-	-	-	-	-
2.05	1.89	1.43	2.02	1.92	1.42	1.94	1.86	1.47
2.05	1.78	1.4	2.02	1.73	1.41	2.03	1.77	1.36
1.79	1.7	1.34	1.81	1.64	1.33	1.85	1.57	1.35
2.06	1.87	1.41	1.99	1.89	1.48	2.04	1.87	1.43
1.45	1.75	1.2	1.47	1.65	1.25	1.43	1.75	1.24
1.87	1.89	1.38	1.78	1.94	1.44	1.88	1.84	1.41
1.96	1.85	1.36	1.93	1.89	1.36	1.9	1.88	1.36
2.15	1.89	1.39	2.18	1.86	1.37	2.15	1.83	1.4
1.95	2	1.55	1.95	1.96	1.6	1.95	1.93	1.59
2.03	1.97	1.57	2.07	1.91	1.63	2.01	1.96	1.62
2.56	2.47	2.14	2.59	2.44	2.12	2.58	2.42	2.1

2.11	1.64	1.28	2.03	1.62	1.34	2.11	1.6	1.31
2.04	1.83	1.49	1.94	1.76	1.5	1.98	1.75	1.56
2	2.03	1.66	2.07	1.96	1.58	2.01	2.01	1.63
1.62	1.35	1.12	1.56	1.38	1.14	1.55	1.38	1.16
1.79	1.62	1.38	1.85	1.64	1.36	1.79	1.62	1.38
1.24	1.49	1.07	1.21	1.52	1.08	1.22	1.53	1.11
2.04	1.79	1.34	2.06	1.77	1.24	2.05	1.77	1.3
1.38	1.59	1.2	1.5	1.47	1.12	1.5	1.58	1.1
1.57	1.74	1.3	1.59	1.66	1.28	1.57	1.7	1.27
1.5	1.76	1.48	1.52	1.72	1.39	1.37	1.72	1.45
1.29	1.72	1.5	1.38	1.66	1.42	1.39	1.69	1.39
1.52	1.81	1.5	1.61	1.76	1.44	1.53	1.84	1.49
1.47	1.75	1.34	1.49	1.76	1.34	1.49	1.73	1.35

r_mean_R*	R0^Est_R*	R0^Sim_R*	R0^Sim_R*	R0^Sim_R*	R0^Sim_R*	R0^Sim_R*	R0^Sim_R*	R0^Sim_R*
1.99	2.63	2.19	2.41	2.15	2.18	2.55	2.25	2.17
1.65	2.43	1.85	2.36	2.07	1.79	2.41	2.01	1.87
1.89	2.74	1.96	2.83	2.26	2.02	2.86	2.23	2.01
1.79	2.63	1.94	2.57	2.19	1.91	2.55	2.26	1.98
1.86	2.44	2	2.16	1.93	1.98	2.08	1.97	2.04
1.61	2.43	1.9	2.54	2.11	1.86	2.36	2.15	1.74
1.16	1.99	1.26	1.98	1.77	1.28	1.96	1.82	1.3
1.36	1.81	1.56	1.89	1.54	1.55	1.92	1.56	1.55
1.03	1.36	1.18	1.09	1.17	1.12	1.16	0.98	1.18
1.12	1.73	1.27	1.96	1.52	1.26	2.04	1.61	1.31
1.25	1.73	1.52	2.12	1.63	1.49	2.11	1.64	1.47
1.28	2.03	1.58	2.21	1.7	1.46	2.27	1.68	1.47
1.15	1.74	1.28	1.81	1.6	1.35	1.87	1.61	1.31
1.88	2.19	2.33	2.24	2.14	2.31	2.3	2.16	2.37
1.76	2.13	2.13	2.16	2.11	2.08	2.18	2.08	2.1
1.34	1.68	1.59	1.52	1.48	1.57	1.59	1.57	1.6
1.29	1.61	1.5	1.47	1.38	1.43	1.54	1.43	1.49
1.2	1.52	1.41	1.49	1.27	1.38	1.46	1.3	1.44
1.22	1.52	1.44	1.37	1.31	1.44	1.45	1.3	1.41
1.08	1.39	1.23	1.04	1.05	1.28	0.94	1.07	1.28
1.1	1.33	1.29	0.94	0.94	1.29	0.91	0.89	1.27
2.04	2.48	2.34	2.49	2.29	2.36	2.52	2.27	2.38
2.01	2.39	2.35	2.41	2.24	2.34	2.43	2.16	2.42
2.27	2.98	2.42	2.77	2.38	2.42	2.83	2.29	2.45
1.78	2.33	2.14	2.49	2.19	2.13	2.53	2.18	2.13
2.4	2.73	2.55	2.3	1.85	2.56	2.4	1.88	2.52
2.13	2.59	2.18	2.2	1.89	2.25	2.26	1.83	2.27
2.53	3.48	2.7	3.46	3.39	2.68	3.51	3.41	2.7
2.39	3.19	2.56	3.23	3.14	2.63	3.13	3.12	2.61
2.13	3.1	2.3	2.92	2.86	2.18	2.96	2.95	2.39
2.57	3.65	2.68	3.47	3.46	2.61	3.48	3.45	2.5
2.74	3.69	2.99	3.63	3.51	2.75	3.57	3.42	2.93
-	-	-	-	-	-	-	-	-
2.38	2.68	2.39	2.2	1.55	2.42	2.14	1.53	2.32
2.47	2.57	2.41	2.06	1.52	2.42	2.01	1.52	2.48
2.01	2.16	2.18	1.93	1.44	2.19	1.95	1.47	2.12
2.51	2.72	2.45	2.07	1.54	2.5	2.1	1.49	2.5
1.8	2.02	1.77	2	1.39	1.8	1.98	1.35	1.8
2.17	2.38	2.24	2.21	1.4	2.23	2.2	1.49	2.2
2.25	2.5	2.38	2.08	1.46	2.29	2.12	1.44	2.33
2.43	2.51	2.62	2.03	1.49	2.56	2.08	1.46	2.57
2.21	2.42	2.45	2.26	1.59	2.45	2.24	1.63	2.42
2.41	2.68	2.51	2.18	1.65	2.52	2.23	1.64	2.55
3.23	3.39	3.21	2.93	2.4	3.24	2.94	2.4	3.23

1.96	1.99	2.48	1.81	1.42	2.46	1.91	1.35	2.51
1.91	1.99	2.38	2.15	1.66	2.36	2.16	1.73	2.33
2.38	2.69	2.46	2.33	1.7	2.4	2.34	1.7	2.48
1.48	1.64	1.76	1.58	1.3	1.89	1.6	1.22	1.84
1.86	1.96	2.18	1.89	1.42	2.16	1.92	1.43	2.19
1.38	1.58	1.57	1.71	1.18	1.44	1.7	1.15	1.45
2.25	2.33	2.5	1.92	1.29	2.41	1.9	1.36	2.42
1.51	1.85	1.84	1.77	1.25	1.77	1.76	1.27	1.73
1.52	1.72	1.92	1.91	1.31	1.94	1.88	1.35	1.98
1.42	1.76	1.97	1.9	1.52	1.86	1.95	1.57	1.85
1.31	1.63	1.68	1.85	1.51	1.63	1.88	1.55	1.67
1.45	1.83	1.92	2.01	1.68	1.77	2.06	1.64	1.96
1.43	1.74	1.8	2	1.47	1.92	1.99	1.49	1.88

R0^Sim_R*	R0^Sim_R*=4_Bursty_g=2
2.46	2.2
2.44	2
2.7	2.35
2.46	2.18
2.15	1.86
2.52	2.17
1.93	1.82
1.76	1.59
1.09	1.02
1.98	1.52
1.99	1.66
2.25	1.81
1.76	1.67
2.3	2.07
2.15	2.15
1.6	1.56
1.58	1.41
1.38	1.26
1.36	1.28
1.03	1.06
0.94	0.91
2.54	2.22
2.42	2.15
2.78	2.32
2.5	2.24
2.41	1.85
2.34	1.81
3.55	3.39
3.11	3.13
2.93	2.91
3.48	3.45
3.59	3.53
-	-
2.17	1.66
2.02	1.48
2.01	1.49
2.13	1.48
1.95	1.43
2.24	1.47
2.19	1.49
2.07	1.49
2.3	1.69
2.19	1.59
2.92	2.35

1.83	1.4
2.17	1.73
2.32	1.7
1.55	1.25
1.84	1.48
1.73	1.25
1.95	1.32
1.7	1.27
1.84	1.34
1.93	1.58
1.88	1.53
1.96	1.66
1.89	1.43
